# A Population-Based Cross-Sectional Investigation of COVID-19 Hospitalizations and Mortality Among Autistic People

**DOI:** 10.1007/s10803-025-06844-6

**Published:** 2025-05-21

**Authors:** Dewy Nijhof, Filip Sosenko, Daniel Mackay, Michael Fleming, Bhautesh D. Jani, Jill P Pell, Chris Hatton, Deborah Cairns, Angela Henderson, Laura McKernan Ward, Ewelina Rydzewska, Maria Gardani, Elliot Millington, Craig Melville

**Affiliations:** 1 School of Health and Wellbeing, University of Glasgow, Glasgow, UK; 2 Faculty of Health and Education, Manchester Metropolitan University, Manchester, UK; 3 School of Health in Social Science, University of Edinburgh, Edinburgh, UK; 4 Health Informatics Centre, School of Medicine, Ninewells Hospital, University of Dundee, Dundee, DD1 9SY UK; 5 General Practice and Primary Care, School of Health and Wellbeing, University of Glasgow, Glasgow, UK

**Keywords:** Autism spectrum disorder, ASD, COVID-19, Morbidity, Mortality, Population study

## Abstract

Current evidence suggests the possibility that autistic people may be at more risk of COVID-19 infection, hospitalisation, and mortality than the general population. Previous studies, however, are either limited in scale or do not investigate potential risk factors. Research into risk factors focused on general population samples. The current study aims to investigate these risk factors in the autistic population. Using data-linkage and a whole-country population, this study modelled associations between autism and COVID-19 hospitalisation and mortality risk in adults, investigating a multitude of clinical and demographic risk factors. Autistic adults had higher rates of hospitalisation, Standardised Incident Ratio 1.6 in 2020 and 1.3 in 2021, and mortality, Standardised Mortality Ratio 1.52 in 2020 and 1.34 in 2021, due to COVID-19 than the general population. In both populations, age, complex multimorbidity and vaccination status were the most significant predictors of COVID-19 hospitalisation and mortality. Effects of psychotropic medication varied by class. Although similar factors exhibited a positive association with heightened risk of severe COVID-19 in both the autistic and general populations, with comparable effect sizes, mortality rates were elevated among the autistic population compared to the general population. Specifically, complex multimorbidity and classification of prescribed medications may emerge as particularly significant predictors of severe COVID-19 among individuals within the autistic population due to higher prevalence of complex multimorbidity in the autistic population and variability in the association between medication classes and severe COVID-19 between both populations, though further research is needed.

## Introduction

In the UK, the earliest COVID-19 cases were identified in late January 2020 and a pandemic was officially declared in March 2020 (GOV.UK, n.d.). Increasing evidence suggests increased risk of susceptibility and severe disease progression of COVID-19 for certain conditions (Li et al., 2021; Zhou et al., 2021). For example, conditions such as diabetes and obesity were deemed risk factors (Zhou et al., 2021). Subgroups within the population have been shown to be disproportionately at risk of COVID-19 but the full extent of these risk factors is unclear (Constantino et al., 2020). Most research has focused on ethnic subpopulations or high-risk patient groups, but little is known about the risks of COVID-19 for autistic people.^1^

Autism Spectrum Disorder (ASD) is defined as a neurodevelopmental disorder. Its main characteristics are differences in social communication and interaction and repetitive and restrictive behaviours, including differences in sensory preferences (American Psychiatric Association, 2013). Whilst several genetic and immunological characteristics of autism have been hypothesised to affect COVID-19 infection, severity, and mortality risk (Brondino et al., 2021; Croonenberghs et al., 2002; Lima et al., 2020), few studies have focused on differences in the clinical profiles of autistic people and the general population. Both physical and mental health conditions, such as obesity (Li et al., 2021; Zhou et al., 2021), gastrointestinal symptoms (Li et al., 2021) and mental health conditions (Toubasi et al., 2021; Vai et al., 2021a) have been associated with increased COVID-19 infection, disease severity and mortality in the general population. Several of these conditions often co-occur alongside autism (Hossain et al., 2020; Rydzewska et al., 2021). Specifically, psychotic disorders and mood disorders are more commonly experienced by autistic people (Hossain et al., 2020) and have been linked with increased risk of COVID-19 mortality in the general population (Toubasi et al., 2021; Vai et al., 2021b). It is further argued by some that sensory difficulties experienced by autistic people can hinder their ability to adhere to COVID-19 measures, such as wearing personal protective equipment (PPE) or strict hygiene rules, thereby increasing the potential for exposure to sources of infection (Eshraghi et al., 2020; Mutluer et al., 2020). Similarly, differences in communication and social isolation can impact the ability to understand and follow COVID-19 related advice and affect help-seeking behaviours and patient-clinician communication in relation to health (Constantino et al., 2020; Eshraghi et al., 2020; Mutluer et al., 2020)**.**

Furthermore, long-term conditions and multimorbidity prevalence is often accompanied by polypharmacy, the use of multiple medicines, which has been shown to contribute to increased COVID-19 morbidity in the general population (Iaccarino et al., 2020; McKeigue et al., 2021) but has not been investigated in autistic populations. Yet, polypharmacy and more particularly prescription of antipsychotics are prevalent both amongst autistic children (Spencer et al., 2013) and autistic adults (Lake et al., 2012; Vohra et al., 2016). Commonly prescribed medications, including antipsychotics, antidepressants, and anticholinergic medication, are known to have the potential to increase the risk of pneumonia through immunosuppressive and other mechanisms (Laporte & Healy, 2020). Moreover, antipsychotics have been shown to exert anti-inflammatory effects and disrupt immune responses (May et al., 2020), which may contribute even further to adverse COVID-19 outcomes. Indeed, several studies (Poblador-Plou et al., 2020; Reilev et al., 2020) and a meta-analysis (Cantudo-Cuenca et al., 2021) found increased risk of COVID-19 mortality associated with exposure to antipsychotics in the general population. Furthermore, recent research has raised the issue of potential adverse outcomes due to drug-drug interactions of prescribed medications and COVID-19 pharmacotherapies (Cantudo-Cuenca et al., 2021; Plasencia-García et al., 2021).

Few studies have investigated the association between autism and COVID-19 and results are inconsistent. One study investigating symptomatic profiles and vaccination side effects in 36 autistic children in a residential facility did not find higher prevalence nor severity of COVID-19 in autistic young adults, though it should be mentioned that study controls (n = 35) were of older age than the autistic group (Brondino et al., 2021)**.** Additionally, previous research suggests that facilities in which individuals live in close proximity have been found to increase infection rates (Gardner et al., 2020) and pose a risk factor for COVID-19 in these populations (Schott et al., 2022). Further, this study did not differentiate between young adults with autism only and young adults with co-occurring autism and intellectual disabilities (ID). Previous research suggests that people with ID are at higher risk of severe COVID-19 (Henderson et al., 2022; Sosenko et al., 2023) and risk factors can have differential impact on autistic adults without ID, ID population and populations with co-occurring autism and ID due to variations of reliance on outside care services, living arrangements and the prevalence of comorbidities across these populatrions. (Schott et al., 2022).

A meta-analysis found increased risk of COVID-19 mortality in autistic people only in unadjusted estimates and no evidence of increased intensive care admissions, but did not separate autism from ID or other developmental disorders (Vai et al., 2021b)**.** In contrast, a population-based study found that autistic males over 16 years had higher COVID-19 hospitalisation rates after stratifying for age, gender and morbidities that have been shown to increase COVID-19 infection rates compared to the general population (Krieger et al., 2023)**.** Moreover, both of these used data from the early pandemic with some inclusion of the initial months of vaccine availability, thus the full effect of vaccinations on COVID-19 infection in autistic populations is still unclear.

As such, there is conflicting evidence regarding the risk of COVID-19 infection and outcome severity amongst autistic people. To the best of our knowledge, no studies have investigated whether this risk extends to COVID-19 mortality in an autistic-only population. Moreover, whilst previous studies speculated on the underlying causes of increased risk, none have systematically studied clinical risk factors for COVID-19 in the autistic population. Using a country-wide sample, the current study aimed to investigate whether there are differences in COVID-19 hospitalisation and mortality rates between autistic people and the general population. Furthermore, this study aimed to explore the association between demographic and clinical risk factors and severe COVID-19 in the autistic population.

## Methods

### Study Design

This was a cross-sectional study using a whole-country population. Data was subdivided into two time periods: (1) including the month in which the first lockdown started in the UK, 1 March 2020, to 31 December 2020; (2) 1 January 2021 to 31 December 2021. This division was made due to the vaccination programme not coming into full effect until 2021. While the first vaccination was received on 8 December 2020, due to vaccination priorities and logistics it is reasonable to assume that the majority of the study population had not received a COVID-19 vaccination in 2020. However, by the end of 2021, a greater proportion of the population had likely been afforded the opportunity to receive at least one COVID-19 vaccination. Consequently, COVID-19 rates in 2021 may vary significantly from those in 2020.

### Data Sources and Definitions

This study was conducted on behalf of the CVD-COVID-UK/COVID-IMPACT Consortium (British Heart Foundation Data Science Centre, n.d.) (coordinated by the BHF Data Science Centre). The following datasets were used: GPES Data for Pandemic Planning and Research (GDPPR), Hospital Episode Statistics (HES), COVID-19 Second Generation Surveillance System (SGSS), Vaccination Events (VE), Civil Registry Deaths from the ONS (ONS-D), NHS BSA Dispensed Medicine (NHS BSA). The GDPPR, HES, SGSS, VE and NHS BSA datasets are updated through the National Health Service (NHS) and processed by NHS England, after which the data is available for secondary uses, such as research. The ONS dataset is updated through the Office for National Statistics (ONS). Approvals were obtained from the research consortium and from the University of Glasgow Ethics Committee.

Long-term conditions (LTCs) were identified using a list of 35 common conditions in the general UK population (Cassell et al., 2018) using phenotypes sourced from the Cambridge Multimorbidity Score, using the GDPPR and HES datasets (Public Health England., 2020)**.** This list includes conditions that have previously been identified as risk factors for COVID-19 infection and severity, for example obesity, diabetes, and hypertension (Li et al., 2021; Zhou et al., 2021)**.** The list includes both physical and mental conditions but excludes autism. Complex multimorbidity was defined by the presence of at least three of those 35 LTCs of which at least one was physical (Kinnear et al., 2018). Polypharmacy was defined as the concurrent use of five prescription medications or more (Masnoon et al., 2017). Polypharmacy, use of antipsychotics, antidepressants, anticonvulsants, and psychotropic medication were identified using British National Formulary (BNF) codes. Prescribing data was examined if it fell within the following parameters: dispensed within the 240 days leading up to the cut-off date (the date of testing positive for COVID-19) and up to 15 days before this cut-off date (Masnoon et al., 2017)**.**

In the context of this study, severe COVID-19 included those who had a positive COVID-19 laboratory test and were hospitalised for COVID-19 or died from COVID-19 within 28 days from the test or died without a positive test, but COVID-19 is listed as a cause of death on their death certificate (any diagnostic position).

### Population

The study covered the whole adult (i.e., over 18 years old) population of England who were alive on 1 January 2020 and had at least one primary care record (for the purpose of establishing their autism status). Autistic individuals were identified using SNOMED CT diagnosis codes in the primary care dataset (GDPPR). As GDPPR is a data extract specifically developed for the purpose of investigating the COVID-19 pandemic (NHS Digital, n.d.), it contains limited historical information about patients. While the coverage of medical history after mid-2018 is strong, the number of data points is much lower prior to then.^2^ When determining the patients’ ASD and ID status, we decided to use all past information about them that was available. However, as a sensitivity analysis we carried out a parallel exercise where only information from mid-2018 onwards was used. That analysis did not suggest that including earlier medical history had a biasing effect.

Existing evidence shows that people with ID have a larger risk of severe COVID-19 (Henderson et al., 2022; Sosenko et al., 2023). Further, research suggests that there may be complex interactions at play in relation to mortality and causes of mortality when it comes to comorbid autism-ID (Forsyth et al., 2023; Schott et al., 2022). As such, this study opted to investigate severe COVID-19 in autism only. Participants who had diagnoses for both autism and ID were included in the comparison group to control for potential confounding (having ID is a major risk factor for COVID-19 hospitalisation or death (Henderson et al., 2022; Schott et al., 2022; Sosenko et al., 2023). Indeed, the crude COVID-19 mortality rate among people with autism and ID (1 in 758) was much closer to that among people with ID (1 in 181) than the one among people with autism only (1 in 6297). Although there is a possibility that placing people with autism and ID in the comparison group may marginally reduce any differences found between autistic people and the comparison group, we maintain the stance that as a result of this decision, any inferences drawn from this study will be more generalisable to autistic people without intellectual disabilities ‒which remains the main focus of the study. ID was identified in an analogical way to autism, that is via SNOMED CT codes in GDPPR. Of all people with autism, approximately 16.5% also had a diagnosis of ID in their primary care records.

The whole population consisted of 180,860 (of which 28.5% female) autistic people and 48,383,120 (of which 50.4% female) people in the comparison group, hereafter referred to as general population. Age distribution in the two populations differed significantly, with a median age of 47 [interquartile range (IQR) 30] years in the general population and 26 [IQR 12] in the autistic population (t-test, *p*-value < 0.001). This corresponds to known low levels of identification or recording of autism in primary care records for people aged over 30 years old (Mehta & Glover, 2019) as well as diagnostic advances and autism awareness over the years (Russell et al., 2022). Demographic and health characteristics of the study participants are shown in Table 1*.* Characteristics for the 35 long-term conditions for both the whole population and analysis sample- (i.e., those infected with and hospitalised for COVID-19) can be found in the *Appendix*.

**Table 1:** Demographic and health characteristics of autistic and general population people hospitalised with a registered positive COVID-19 test

	General population (n = 8,425,470)	Autistic population (n = 32,375)			
		Adjusted	P-value	Unadjusted	P-value
Age [Mdn., IQR]	41.8 [30.0, 55.0]	–	–	24.9 [21.0, 31.5]	< 0.001
Female (%)	54.0	33.7	< 0.001	33.4	< 0.001
Mean IMD decile (SD)	5.4 (2.9)	5.0 (2.8)	< 0.001	5.1 (2.9)	< 0.001
Ethnicity (%)			< 0.001		< 0.001
White	82.7	93.5		92.0	
Asian	9.2	2.2		2.4	
Black	3.7	1.6		2.0	
Multiracial	1.9	1.6		2.4	
Other	2.4	1.1		1.3	
Vaccine			0.8		< 0.001
AstraZeneca	21.2	23.4		30.1	
Moderna	3.4	3.1		7.6	
Pfizer	26.4	24.4		62.2	
Vaccine doses			> 0.9		< 0.001
0	49.3	49.1		50.8	
1	18.3	18.4		19.4	
2	32.5	32.5		29.8	
Total number of prescribed medications			< 0.001		< 0.001
0	55.8	36.1		53.4	
1–3	23.0	24.4		25.8	
4–6	9.3	14.5		10.4	
7–9	4.9	9.0		4.9	
10 +	7.0	16.1		5.4	
Mean prescription medications (SD)	2.9 (4.5)	5.1 (6.0)	< 0.001	2.7 (3.6)	0.002
Polypharmacy (%)	21.2	39.5	< 0.001	20.8	0.14
Medications split by class			< 0.001		–
On Psychotropics (%)					
No	80.9	49.7		–	
Yes	19.1	50.3		–	
Antipsychotics (%)	1.3	12.7	< 0.001	–	–
Antidepressants (%)	17.1	45.1	< 0.001	–	–
Anticonvulsants (%)	3.9	11.7	< 0.001	-	–
Morbidity					
Mean LTCs (SD)	1.0 (3.0)	2.4 (4.0)	< 0.001	1.2 (2.7)	< 0.001
Multimorbidity (%)	11.9	24.3	< 0.001	12.4	< 0.001

From this whole population, participants were included in the analysis if they met at least one of two criteria: (1) they had a registered positive COVID-19 laboratory test (identified through GDPPR) or (2) COVID-19 was listed as a cause of death (either primary or other contributing causes using ICD-10 code U07.1 or U07.2) on their death certificate. In the case of multiple positive tests, the first instance was used. Information about LTCs, vaccination status, and prescribed medications were extracted using the time of positive test result.

### Data Analysis

Data was analysed within NHS England’s Secure Data Environment service for England, made available via the British Heart Foundation Data Science Centre’s CVD-COVID-UK/COVID-IMPACT Consortium (British Heart Foundation Data Science Centre, n.d.), using a combination of Python (v3.7), SQL, and R (v4.03) scripts. The analysis was performed according to a pre-specified analysis plan published on GitHub (Github Analysis Plan, n.d.), along with the phenotyping and analysis code.

Demographic (age, sex, ethnicity, Index of Multiple Deprivation (IMD)) and health characteristics (LTCs, complex multimorbidity, vaccination status, prescribed medications) were explored and compared using means, standard deviations, and t-tests for continuous data and frequency, percentages, and χ^2^-tests for categorical data. Standardised Incidence Ratios (SIRs) and Standardised Mortality Ratios (SMRs) were calculated for 2020 and 2021 separately. SIRs were also calculated for hospitalisation (while positive for COVID-19) and severe COVID-19 (hospitalisation or death due to COVID-19). A binary logistic regression model was used to model prediction of severe COVID-19 infection using demographic and health factors as predictors. To avoid interpretational difficulties resulting from having many interactions, the model was fit separately to the autistic and non-autistic populations. The only interaction tested in the model was the one between multimorbidity and polypharmacy, since we thought is possible that the effect of the latter varies by the level of the former. Note that modelling of antipsychotic, antidepressant, and anticonvulsant medication did not control for diagnosis of related mental health conditions due to the possibility of prescription of these medications for unrelated issues. There is evidence to suggest that overprescription, particularly of psychotropic medications, is a significant issue in relation to autistic adults (Glover et al., 2015; Mehta & Glover, 2019) with and without psychiatric conditions (Carthy, 2021). Further, though research on these medication classes in autistic people is scarce, limited evidence suggests variable effects regarding efficacy and tolerance of psychotropic medication in autistic people (Carthy et al., 2021).

Further hierarchical logistic modelling was used to assess the contribution of each predictor category. The order in which predictors were added was determined by the following strategy: Demographic factors, such as age, were added before health-related factors (e.g. vaccination status). Within each category of factors, predictors were ordered by the assumed strength of the effect on the outcome, based on existing knowledge about COVID-19. The resulting order of adding predictors was as follows: age, sex, ethnicity, area deprivation, vaccination status, multimorbidity, and finally polypharmacy.

Blinder-Oaxaca decomposition analysis was applied to decompose the mean difference in the risk of severe COVID-19 between the two population groups for each of the predictor categories used in the regression modelling (Rahimi & Hashemi Nazari, 2021).

Logistic regression was chosen as the main modelling technique rather than survival analysis due to its allowance for a larger number of records to be included. (Individuals who had a PCR test already in hospital, and those who died without a PCR test, cannot be included in survival analysis). Still, survival analysis in the form of a Cox proportional hazards model was conducted as a sensitivity test of the logistic modelling.

## Results

### Demographics

A total of 32,375 autistic adults and 8,425,470 general population adults were hospitalised with a positive COVID-19 test. Table 1 shows the demographic and health characteristics of autistic people and the general population used in the analysis (i.e., tested positive for COVID-19). Due to the younger age structure in the autistic population, both unadjusted and age-adjusted values are shown. The autistic group had a significantly different population structure, demonstrated by a lower percentage of females, higher chance of living in a deprived area (evidenced by lower mean IMD decile) and a higher percentage of white ethnicity. Autistic adults were equally likely as the general population to be vaccinated for COVID-19. Polypharmacy was more prevalent in the autistic population, with the mean numbers of prescription medications being 5.1 (SD 6.0) compared to 2.9 (SD 4.5) for the general population. Similarly, use of psychotropic, antipsychotic, antidepressant and anticonvulsant medications, was higher within the autistic population.

Autistic adults were also more likely to experience complex multimorbidity. From the list of 35 most common conditions in the UK, only three were not significantly higher for autistic adults (bronchiectasis, chronic kidney disease, and multiple sclerosis; see Appendix for full list). A few conditions stood out due to particularly higher prevalence in the autistic population: stroke/transient ischaemic attack, schizophrenia/bipolar disorder, psychoactive substance misuse, Parkinson’s disease, diabetes, depression, dementia, asthma, anxiety/other neurotic disorders, and anorexia/bulimia.

### Incidence, Mortality and Survival Analysis

Table 2 shows the SIRs for autistic adults who were hospitalised due to COVID-19 or who classify as having severe COVID-19 (i.e., taking together COVID-19 hospitalisations and COVID-19 deaths). The ratios show a higher incidence of both hospitalisation and severe COVID-19 for autistic adults in comparison to the general population, although a decline over time is visible from 2020 to 2021.

**Table 2:** Standardised Incidence Ratios for autistic adults with general population as reference

	COVID-19 hospitalisation				Severe COVID-19			
	Observed	Expected	Excess	SIR [95% CI]	Observed	Expected	Excess	SIR [95% CI]
Mar–Dec 2020	372	234	138	1.6 [1.4, 1.7]	382	244	138	1.6 [1.4, 1.7]
Jan–Dec 2021	603	461	142	1.3 [1.2, 1.4]	620	469	151	1.3 [1.2, 1.4]

Table 3 shows the Age Standardised Mortality Rate (ASMR) and Standardised Mortality Rates (SMR). ASMR takes the European Standard Population 2013 as reference and SMR takes the general population from the study sample as reference. Whilst ASMRs were higher for both groups within the study sample, the autistic population had particularly high mortality rates compared to the European Standard Population. Within the study sample, mortality rates for autistic adults were similarly increased compared to the general population. Similar to incidence rates, a decline from 2020 to 2021 is visible.

**Table 3:** Age Standardised Mortality Rates (ASMR) for the adult autistic and general population in study sample as compared to the European Standard Population 2013. Second column shows Standardised Mortality Ratios (SMR) for the autistic population as compared to the general population for the study sample only. Observed, expected and excess mortality for SMRs are shown

	ASMR [95% CI]		SMR [95% CI]			
	Autistic population	General population	Autistic population	Observed	Expected	Excess
Mar–Dec 2020	278.9 [278, 278]	166.2 [166, 166]	1.52 [1.04, 2.0]	39	25.6	13.4
Jan–Dec 2021	271.5 [271, 271]	143.1 [143, 143]	1.34 [0.93, 1.74]	42	31.5	10.5

### Modelling of Risk Factors

Logistic regression modelling has been employed to investigate risk factors. The final model included two versions sharing core predictor variables and differed only in the aspect of medications. Model A focused on polypharmacy while model B focused on psychotropic medication.

Table 4 shows the results from the logistic regression models A and B. For both the autistic and general population, several factors were positively associated with increased risk of severe COVID-19 with similar effect sizes: age, ethnicity, complex multimorbidity (count of long-term conditions), polypharmacy (count of prescription medication), and antipsychotic prescription. Interestingly, the effect of complex multimorbidity varies by the extent of polypharmacy and vice versa. The effect of either risk factor is stronger when the score for the other is low. Again, effect sizes were similar between the two groups. As the demographic descriptives showed, the autistic population had an overall younger age structure than the general population, yet age effect sizes in the logistic and hierarchical regression models were similar between the two groups. Blinder–Oaxaca Decomposition analysis further confirmed that the difference in non-age-adjusted probability of severe COVID-19 (0.066 in the general population, 0.033 in the autism group) can largely be attributed to the younger age profile of adults with autism (see Appendix).

**Table 4:** Results from Logistic Models A and B fit to the autistic and general Populations separately

Predictors	Model A				Model B			
	General population		Autistic population		General population		Autistic population	
	Coefficient [95% CI]	*P*-value	Coefficient [95% CI]	*P*-value	Coefficient [95% CI]	*P*-value	Coefficient [95% CI]	*P*-value
Age at COVID-19	1.039 [1.039, 1.039]	<.001	1.037 [1.031, 1.042]	<.001	1.044 [1.044, 1.044]	<.001	1.038 [1.033, 1.043]	<.001
Female	0.713 [0.705, 0.720]	<.001	0.729 [0.570, 0.888]	<.001	0.756 [0.749, 0.764]	<.001	0.808 [0.648, 0.968]	.01
IMD decile	0.969 [0.968, 0.971]	<.001	1.002 [0.976, 1.029]	.86	0.964 [0.963, 0.965]	<.001	-	-
Ethnicity								
White	(base)							
Asian	1.770 [1.758, 1.781]	<.001	1.695 [1.280, 2.109]	.01	1.798 [1.786, 1.809]	<.001	1.587 [1.173, 2.001]	.03
Black	2.162 [2.145, 2.179]	<.001	1.468 [1.009, 1.926]	.10	2.075 [2.058, 2.091]	<.001	1.313 [0.856, 1.770]	.24
Multiracial	1.654 [1,625, 1.683]	<.001	1.253 [0.798, 1.709]	.33	1.622 [1.594, 1.650]	<.001	1.279 [0.830, 1.729]	.28
Other	2.119 [2.096, 2.142]	<.001	1.372 [0.755, 1.990]	.32	2.005 [1.982, 2.027]	<.001	1.362 [0.749, 1.975]	.32
Vaccine								
Number of vaccine doses	0.740 [0.724, 0.755]	<.001	0.724 [0.370, 1.077]	.07	0.747 [0.731, 0.763]	<.001	0.730 [0.367, 1.093]	.09
AstraZeneca	0.489 [0.459, 0.520]	<.001	0.539 [−0.149, 1.227]	.07	0.465 [0.434, 0.497]	<.001	0.508 [−0.199, 1.216]	.06
Pfizer	0.297 [0.273, 0.321]	<.001	0.268 [−0.291, 0.828]	<.001	0.285 [0.260, 0.310]	<.001	0.253 [−0.323, 0.829]	<.001
Moderna	0.122 [0.062, 0.182]	<.001	0.203 [-.0733, 1.139]	.001	0.118 [0.058, 0.178]	<.001	0.187 [−0.765, 1.139]	.001
Interactions								
Count of long-term conditions	1.550 [1.548, 1.551]	<.001	1.570 [1.543, 1.597]	<.001	1.370 [1.369, 1.370]	<.001	1.436 [1.419, 1.453]	<.001
Count of prescription medicines	1.101 [1.100, 1.102]	<.001	1.084 [1.057, 1.111]	<.001	–	–	–	–
Interaction of the above two counts	0.984 [0.984, 0.984]	<.001	0.985 [0.982, 0.988]	<.001	–	–	–	–
Prescription medication by class								
Antipsychotic	-	–	–	–	1.713 [1.693, 1.733]	<.001	1.262 [1.044, 1.479]	.03
Antidepressant	-	–	–	–	0.867 [0.857, 0.876]	<.001	0.643 [0.475, 0.811]	<.001
Anticonvulsant	-	–	–	–	1.082 [1.068, 1.095]	<.001	1.028 [0.805, 1.250]	.81
Constant	0.005 [−0.010, 0.020]	<.001	0.004 [−0.252, 0.260]	<.001	0.006 [−0.009, 0.020]	<.001	0.006 [−0.201, 0,.214]	<.001
Observations	8,259,596	–	31,758	–	8,259,596		31,782	

Modelling COVID-19 diagnosis by age (at diagnosis), sex, IMD decile, ethnicity (white ethnicity used as base), vaccine brand, vaccine dose count, long-term condition count. Model A additionally includes prescription medication count and the interaction between long-term condition count and prescription medication count. Model B additionally includes use of antipsychotic medication, antidepressant medication, and anticonvulsant medication

Anticonvulsant prescription was significantly but weakly positively associated with increased risk for the general population only. Whereas Asian/Asian British ethnicity was the only ethnicity at risk for the autistic population, Black/Black British ethnicity was most at risk for the general population followed by Other, Asian/Asian British and Multiracial ethnicity. Additionally, the effects of ethnicity and antipsychotic prescription on severe COVID-19 risk were more profound for the general population. Effects of long-term condition count were more pronounced for the autistic population. Contrastingly, female sex, Pfizer and Moderna vaccination, and antidepressant prescription were associated with decreased risk of severe COVID-19 for both the autistic and general population. Number of COVID-19 vaccination doses and AstraZeneca vaccination were significantly associated with decreased risk for the general population only. Antidepressant prescription effects were more pronounced for the autistic population. Results from the survival analysis showed similar trends in associations, thus supporting findings from the logistic regression models (see Appendix).

Hierarchical logistic modelling (Table 5) indicated that age, vaccination status and LTC count were the most important predictors for modelling severe COVID-19 infection in both autistic adults and the general population.

**Table 5:** Results from the hierarchical logistic regression showing Pseudo R^2^, Akaike Information Criterion (AIC) and Bayesian Information Criterion (BIC) modelling COVID-19 diagnosis by age, sex, ethnicity, IMD decile, long-term condition count, and polypharmacy

	Autistic population			General population		
	Pseudo R2	AIC	BIC	Pseudo R2	AIC	BIC
Age	0.091	8353	8370	0.232	30,871,388	30,871,666
+ sex	0.093	8340	8365	0.234	30,789,963	30,790,381
+ ethnicity	0.094	8336	8394	0.241	30,524,687	30,525,662
+ IMD decile	0.095	8326	8393	0.248	30,241,212	30,242,327
+ vaccine status	0.145	7880	7989	0.297	28,292,202	28,294,012
+ LTC count	0.224	7160	7277	0.364	25,565,855	25,567,805
+ polypharmacy	0.225	7146	7272	0.369	25,358,599	25,360,689

Vaccine status is defined by the presence or absence of at least one COVID-19 vaccination

## Discussion

This study aimed to explore COVID-19 in autism by investigating hospitalisation and mortality rates and exploring the association between demographic and clinical risk factors and their contributions to severe COVID-19 in autistic people. Previous studies investigating COVID-19 in autistic people produced conflicting results. Similar to a previous population-based study (Krieger et al., 2023), our findings provide further evidence for the increased risk of hospitalisation (SIR 2020: 1.6, SIR 2021: 1.3) and mortality (SMR 2020: 1.52, SMR 2021: 1.34) of autistic adults due to COVID-19.

Exploration of demographic and clinical factors through regression modelling showed similar risk factors for the autistic and general population, with a few exceptions. Whereas all minority ethnicities were predictive of severe COVID-19 for the general population, only Asian/Asian British ethnicity was a significant predictor for the autistic population. This may be due to the different distribution of ethnicity between the two groups, with non-white ethnicities being less prevalent in the autistic population affecting sample size for those ethnicities (see Table 1). Similarly, female sex had a more protective effect for the general population than for the autistic population which may be explained due to the male bias in autism prevalence (Lai et al., 2014).

Number of COVID-19 vaccination doses only differed when not age-adjusted, however, the brands of vaccination had differing effects in the two populations. For both the general and autistic population, most people had received either Pfizer or AstraZeneca. Modelling of the predictors showed that while Pfizer was a significantly protective factor for severe COVID-19 for both populations, AstraZeneca was only significantly protective for the general population. Moderna was received the least by both populations and was found to be the most protective factor out of all predictors. There was no difference in the total number of vaccination doses received by either population.

The number of long-term conditions emerged as an important predictor of severe COVID-19 for both populations, although the results combined suggest a more complex role within the autistic population. While logistic regression models show comparable associations for LTCs in both groups, differences emerge in the proportional hazards model, where the effect of LTCs appears slightly more pronounced in the autistic population for the model focused on antipsychotic medications rather than general prescription medications. Combined with the findings of elevated SMRs and SIRs for the autistic population, this may point to a more nuanced interplay between LTCs and prescription medications, particularly psychotropic medications.

Though the current study did not investigate associations between the individual long-term health conditions and COVID-19 hospitalisation and mortality, it is possible that this finding is due to the different clinical profile of the autistic population. Complex multimorbidity in general but particularly mental health conditions such as depression, dementia, anxiety and schizophrenia were more prevalent in the autistic study population. Previous research identified associations between increased risk of COVID-19 mortality and psychotic disorders, mood disorders and substance use disorders (Toubasi et al., 2021; Vai et al., 2021b). It is plausible that medication regimen, coupled with the presence of multimorbidity, may potentially lead to complex interactions that influence COVID-19 outcomes. Therefore, future research endeavours should aim to untangle the effects of medication regimen and the composition of multimorbidity in the autistic population on COVID-19 outcomes, warranting a separate and focused investigation.

Further, survival modelling showed that similarity in the number of prescriptions and the number of long-term conditions decreased the risk of severe COVID-19. In other words, low values of one variable paired with high values of the other were associated with higher risk of severe COVID-19. This may suggest that well-treated conditions and appropriate use of prescription medications may be a protective factor for severe COVID-19. Although this was found for both the autistic and general population, there is evidence to suggest that the health needs of autistic people are sometimes overlooked (Nicolaidis et al., 2013; Strydom et al., 2021) or exacerbated by barriers in accessing medical care (Jeste et al., 2020; Saqr et al., 2018). Despite similar effect sizes in the two populations, mortality rates were higher for the autistic population. Our findings highlight the importance and potentially far-reaching impact of addressing health inequalities experienced by autistic people.

Whilst polypharmacy was not included amongst the most important predictors in the hierarchical model for either the autistic or general population, further exploration suggests that this might be due to different effects of the medication class. For both the autistic and general population, antipsychotic medication was found to be predictive of severe COVID-19 whereas antidepressant medication was protective. Furthermore, antipsychotic prescription was more predictive of severe COVID-19 in the general population whereas antidepressant prescription was more protective for the autistic population. Potentially, this could be due to differences in how these prescriptions are targeted in the two populations. For example, these medication classes are prescribed to autistic people on a more widespread basis with antipsychotics being prescribed in absence of comorbid psychiatric conditions (Carthy et al., 2021). The finding of protective effects of antidepressant prescription corresponds to prior research suggesting lower infection rates and decreased COVID-19 severity in patients using antidepressants in the general population (Hoertel et al., 2021; Nakhaee et al., 2022). Whilst there are contradictory findings, pooled effects seem to indicate a protective effect of antidepressants against severe COVID-19 (Nakhaee et al., 2022).

Due to abovementioned targeting of prescription medications in the autistic population and potential of prescription medications to be used for a multitude of conditions, our study did not match prescribed medications to existing conditions as this would not be an accurate representation of the use of medications in the autistic population. As such, we are unable to ascertain whether there is a potentially mediating effect between medication and condition on severe COVID-19. For example, several studies provide evidence that psychiatric conditions such as schizophrenia, depression and anxiety have higher COVID-19 mortality rates (Toubasi et al., 2021; Vai et al., 2021b). Results from the hierarchical modelling suggest that the long-term condition is indeed a more significant risk factor than polypharmacy. However, the classes of medication explored in this study can be prescribed for a multitude of conditions and, thus, were not linked to specific long-term conditions. In addition, there are concerns that there may be adverse reactions of prescription medication and COVID-19 treatments (Laporte & Healy, 2020; May et al., 2020). Due to the unavailability of COVID-19 treatment data, this study was unable to ascertain whether prescription medication was taken during the hospital stay and whether there could be potential reactions with COVID-19 treatments provided. Additionally, exploring the effects of differing combinations of prescription medication(s) and long-term conditions was beyond the scope of the current study. However, our results suggest that this may be a viable target for future research.

## Strengths and Limitations

This study has several strengths. The study makes use of a whole-country population of autistic individuals and the general population. This large sample size ensures a representative sample, thereby reducing sampling bias. The combination of the large sample size and the use of validated datasets further strengthens the validity and robustness of the results. Moreover, inclusion criteria for autism are based on clinical diagnoses, using both older and newer diagnosis codes relevant to autism. This allows us to better capture people on the spectrum and, thus, better represent the autistic population in England.

A limitation is that, in earlier stages of the pandemic, community incidence of COVID-19 is likely underreported as testing was not yet standard practice and availability of tests was limited. However, it is likely that testing rates do not differ between people with and without autism. Similarly, case fatality rates may be inflated due to lack of testing in these earlier stages. Additionally, whilst the dataset makes use of a whole-country population dataset, it should be noted that the final merged dataset contained a larger number of records than the recorded population size of England. This may be due to duplication of records, incorrect matching of NHS and death records, or people not registered as living in the UK receiving NHS treatment. However, effects on results reported in this study are likely to be minimal due to its use of rates and ratios, though caution should be exercised when interpreting absolute values.

Due to the use of autism identification through SNOMED CT diagnosis codes through primary care data, it is possible that the autistic study population does not fully capture the autistic population in England. Previous studies have indeed identified gaps in identification of autistic people over 30 years old within primary care (Mehta & Glover, 2019). Moreover, it is possible that some autistic individuals are not registered with primary care and thus were not identified in this dataset.

## Conclusion

To conclude, our results show that autistic adults are at higher risk of hospitalisation or death due to COVID-19 as compared to the general population. Whilst demographic and clinical risk factors are similar for the autistic and general population, our results suggest that complex multimorbidity and prescription medication class may be of particular importance for the autistic population and should be further researched.

## Data Availability

The data used in this study are available in NHS England’s Secure Data Environment (SDE) service for England, but as restrictions apply they are not publicly available NHS England Secure Data Environment. The CVD-COVID-UK/COVID-IMPACT programme led by the BHF Data Science Centre received approval to access data in NHS England’s SDE service for England from the Independent Group Advising on the Release of Data (IGARD) (52) via an application made in the Data Access Request Service (DARS) Online system (ref. DARS-NIC-381078-Y9 C5 K)(NHS Digital DARS). The CVD-COVID-UK/COVID-IMPACT Approvals & Oversight Board (BHF Data Science Centre) subsequently granted approval to this project to access the data within NHS England’s SDE service for England. The de-identified data used in this study were made available to accredited researchers only. Those wishing to gain access to the data should contact bhfdsc@hdruk.ac.uk in the first instance. For the purpose of open access, the authors have applied a Creative Commons Attribution (CC BY) licence to any Author Accepted Manuscript version arising from this submission.

## Footnotes

1 Throughout this paper we use identity-first language or neutral terms (‘autistic people’ or ‘people on the autism spectrum’) rather than person-first language as a result of the article by Kenny et al. (2015) highlighting the preference for identity-first language by the majority of autistic people and their families.

2 https://bhf-dsc-hds.shinyapps.io/cvd-covid-tre-dashboard/_w_381be737/session/ee3189cfcddc0ceca5550982593e074f/download/summary_module-data_coverage_module-download_coverage_data_csv?w=381be737
